# An outbreak of Shiga toxin-producing *Escherichia coli* serotype O103:H2 associated with unpasteurized soft cheese, England and Wales, 2022

**DOI:** 10.1017/S0950268824001523

**Published:** 2025-01-22

**Authors:** Ellen Heinsbroek, Eleanor Blakey, Alex Simpson, Neville Q Verlander, David R. Greig, Frieda Jorgensen, Andrew Nelson, Amy Douglas, Sooria Balasegaram, Claire Jenkins, Richard Elson

**Affiliations:** 1 Field Service East of England, Health Protection Operations, UK Health Security Agency, Cambridge, UK; 2 Gastrointestinal Infections and Food Safety (One Health) Division, Clinical and Public Health Group, UK Health Security Agency, London, UK; 3 Statistics Unit, Statistics, Modelling and Economics Department, UK Health Security Agency, London, UK; 4 Gastrointestinal Bacteria Reference Unit (GBRU), Public Health Microbiology Division, Specialised Microbiology & Laboratories Directorate, UK Health Security Agency, London, UK; 5 Food, Water and Environmental Microbiology Services, Porton Laboratory, UK Health Security Agency, Salisbury, UK; 6 Communicable Disease Surveillance Centre, Public Health Wales, Cardiff, UK; 7 Field Service South East and London, Health Protection Operations, UK Health Security Agency, London, UK

**Keywords:** foodborne infections, food safety, outbreaks, Shiga-like toxin-producing *E. coli*, whole genome sequencing

## Abstract

In July 2022, a genetically linked and geographically dispersed cluster of 12 cases of Shiga toxin-producing *Escherichia coli* (STEC) O103:H2 was detected by the UK Health Security Agency using whole genome sequencing. Review of food history questionnaires identified cheese (particularly an unpasteurized brie-style cheese) and mixed salad leaves as potential vehicles. A case–control study was conducted to investigate exposure to these products. Case food history information was collected by telephone. Controls were recruited using a market research panel and self-completed an online questionnaire. Univariable and multivariable analyses were undertaken using Firth Logistic Regression. Eleven cases and 24 controls were included in the analysis. Consumption of the brie-style cheese of interest was associated with illness (OR 57.5, 95% confidence interval: 3.10–1,060). Concurrently, the production of the brie-style cheese was investigated. Microbiological sample results for the cheese products and implicated dairy herd did not identify the outbreak strain, but did identify the presence of *stx* genes and STEC, respectively. Together, epidemiological, microbiological, and environmental investigations provided evidence that the brie-style cheese was the vehicle for this outbreak. Production of unpasteurized dairy products was suspended by the business operator, and a review of practices was performed.

## Key findings

- An outbreak of 12 geographically distributed but genetically linked cases of Shiga toxin-producing *Escherichia coli* serotype O103:H2 was detected in 2022.
- A case–control study provided evidence that the outbreak was associated with a specific unpasteurized soft cheese product.
- When the link to the unpasteurized cheese was identified, production of unpasteurized dairy products was suspended by the business operator, and a review of practices was performed.
- Results of further microbiological testing of the unpasteurized milk and cheese were satisfactory and no further cases were reported, indicating that it was likely a one-off contamination event had occurred.

## Introduction

In England, raw drinking milk and other unpasteurized dairy products sourced from cows, and to a lesser extent from sheep, goats, and buffalo, can be sold by registered producers directly to the customer at the farm gate or farmhouse catering operation, at farmers’ markets, by distributors using a vehicle as a shop such as a milk round or via direct online sales [1]. Cheese made with raw milk can also be sold via a distributer for general retail sale. Milk can become contaminated from the faeces of infected or colonized animals during milking or from the wider farm environment. As a result, raw drinking milk and other unpasteurized dairy products may have a diverse microbial flora, which can include pathogens transmissible to humans, such as *Brucella melitensis*, *Mycobacterium bovis*, tick-borne encephalitis virus, *Listeria monocytogenes*, and a range of gastrointestinal pathogens, including *Campylobacter* species, *Salmonella* species, and Shiga Toxin-producing *E. coli* (STEC). In the United Kingdom, the National Health Service advises that vulnerable people including those who are pregnant should avoid the consumption of unpasteurized milk and soft cheeses [2].

STEC belong to a pathogenic group of zoonotic *E. coli* that cause gastrointestinal disease in humans due to their ability to produce Shiga toxin (stx) [3]. There are two types of *stx, stx1*, and *stx2*, and 10 subtypes *stx1a, stx1c, stx1d*, and *stx2a-stx2g [4].* The majority of STEC isolated from patients with severe symptoms also have a gene-designated *eae* that encodes the protein intimin, which is required for the formation of attaching and effacing lesions on gut mucosa of the host [5]. STEC infection damages the lining of the gut reducing its capacity to reabsorb fluids resulting in diarrhoea, which may contain blood. Certain strains of STEC have the potential to cause haemolytic uraemic syndrome (HUS), a life-threatening condition characterized by renal failure, sometimes with cardiac and/or neurological complications [6, 7].

Over the last 40 years, the most commonly detected STEC serotype in the United Kingdom was STEC O157:H7, partly because diagnostic laboratory methods focused on the use of cefixime-tellurite sorbitol MacConkey (CT-SMAC) agar selective for the growth of this specific serotype. Over the last 10 years, an increasing number of diagnostic laboratories in England have implemented commercial PCR assays for the detection of gastrointestinal pathogen that target the presence of *stx* and therefore have the potential to detect STEC of all serotypes. As result, there has been a rise in the number of non-O157 STEC serotypes detected isolated from humans with clinical symptoms in recent years.

A wide range of animals can become transiently colonized with STEC, including domestic pets, small mammals, and birds; however, ruminants are the main animal reservoir. In addition to STEC O157:H7, the STEC serotypes most commonly isolated from humans in the United Kingdom are O26:H11, O103:H2, O146:H21, and O91:H14 [8]. All these STEC serotypes are known to be able to colonize the gut of ruminants, specifically cattle, sheep, and goats, and are endemic in the UK ruminant population [9–11]. Transmission to humans occurs following the consumption of contaminated food or water, or via direct contact with animals or their environment. In household and institutional settings, secondary person-to-person transmission of STEC has been described [12].

In June 2022, routine microbiological surveillance at the UK Health Security Agency (UKHSA) identified a cluster of cases of STEC O103:H2 *stx1a/eae* and a multi-agency incident management team (IMT) was convened. This report describes the outbreak investigation, highlights the microbiological and epidemiological challenges encountered and makes recommendations for future practice.

## Methods

### Microbiology investigations

In England, faecal specimens from hospitalized or community cases with symptoms of gastrointestinal disease are routinely cultured in local hospital microbiology laboratories for identification of *Salmonella*, *Campylobacter*, *Shigella* spp., and STEC O157:H7. At the time of this investigation, approximately 20% of laboratories used commercial PCR assays for the detection of gastrointestinal pathogens, including STEC (i.e., also detecting non-O157 STEC) [8]. Faecal specimens from patients where there is a clinical suspicion of HUS and/or testing positive for STEC by PCR and culture-negative for STEC O157:H7 on CT-SMAC agar are submitted to the Gastrointestinal Bacteria Reference Unit (GBRU) at UKHSA for confirmation by PCR and culture [6, 13]. All strains of STEC isolated from faecal specimens were sequenced, and serotype, *stx* subtype profile and single nucleotide polymorphism (SNP) type were derived from the genome, as described previously [6, 13]. A soft-core genome alignment was generated from SnapperDB v0.2.8 [14] of outbreak genomes, this alignment had recombination masked by Gubbins v2.00 [15] and a maximum-likelihood phylogeny was constructed using IQTree v2.0.4 [16].

### Case definitions

Confirmed: A case of STEC O103:H2 *stx1a* +ve and *eae* +ve belonging to the SNP designation 4.12.1120.1519.1597.1612% reported by GBRU since 1 May 2022.

Previous analysis of the relatedness of isolates of STEC has shown that isolates from cases epidemiologically linked to the same outbreak fall within the same five-SNP single-linkage cluster [6, 17].

### Epidemiological investigations

Prospective and retrospective case ascertainment was undertaken by reviewing all whole genome sequencing data held in the UKHSA. UKHSA operates a national enhanced surveillance system for STEC [18]. Public health follow-up focuses on those cases infected with STEC that are positive by PCR for *stx2*, because of the association between *stx2a* and severe clinical outcomes; not all cases that are infected with STEC that are positive for *stx1* only are routinely administered a standardized enhanced surveillance questionnaire (ESQ) [19].

Cases in this outbreak were initially interviewed with an ESQ that collects standardized information on the patient’s food history, contact with animals and environmental exposures for 7 days prior to the onset of illness. Following an initial review of the ESQ data, it was noted that a number of cases reported eating the same artisan cheese, and outbreak cases were re-interviewed using a modified trawling questionnaire to collect a more detailed food history focussed on dairy and salad products.

### Analytical studies

A high proportion of cases mentioned exposure to brie-style cheese, hard cheese, or mixed salad leaves. A case–control study was designed to test the null hypotheses that illness was not associated with exposure to soft, in particular brie-style cheese, hard cheeses, or mixed salad leaves. Confirmed cases were included if they had no history of foreign travel and no close contact with other individuals with diarrhoea in the 7 days prior to onset of illness. Controls were recruited using a market research company, a strategy described previously [20]. Briefly, a market research company has a panel of registered respondents of which a targeted subset is sent a link to an online questionnaire. Respondents who complete the questionnaire are renumerated. Two controls were recruited for each case, frequency-matched on UKHSA region of residence, age group (0–18 years, 19–50 years, and >50 years), and socio-economic status as assessed by the index of multiple deprivation (IMD) based on an individual’s residential postcode [21]. The IMD is the official measure of relative deprivation for at a small local area level in England, based on seven different domains of deprivation: Income Deprivation; Employment Deprivation; Education, Skills and Training Deprivation; Health Deprivation and Disability; Crime; Barriers to Housing and Services; and Living Environment Deprivation [21]. The IMD ranks every small area in England from 1 (most deprived area) to 32,844 (least deprived area). IMD deciles and quintiles are calculated to group the IMD ranking; quintiles were used to frequency-match for this analysis. Exclusion criteria for the control selection were having a diet that excluded the consumption of dairy products, any history of foreign travel in the 7 days prior to completing the questionnaire, and any close contact with any individual with diarrhoea in the 7 days prior to completing the questionnaire.

### Data collection and handling

The questionnaire was administered to cases by phone. Up to three attempts to contact each case by telephone were made, after which the questionnaire was provided by email. Controls recruited by the market research company completed an online questionnaire. Data from interviews and online questionnaires were collected in compliance with data protection guidelines.

### Ethics

UKHSA has delegated authority, on behalf of the Secretary of State, to process Patient Confidential Data under Regulation 3 The Health Service (Control of Patient Information) Regulations 2002. Regulation 3 makes provision for the processing of patient information for the recognition, control, and prevention of communicable disease and other risks to public health.

### Data analysis

Firth’s logistic regression was first used in a series of univariable analyses that modelled the association of each exposure variable with illness by comparing the proportion of exposures reported by cases and controls. Those variables that were possibly associated with illness (*P*-value <0.2 and an odds ratio greater than 1.0) were combined in a multivariable analysis that also controlled for certain demographic variables (age, IMD, region, and gender). Non-significant variables (*P*-value >0.1) that were not substantially confounding were removed from the model in a backwards stepwise procedure. Data were analysed in Stata (v.17, StatCorp).

### Food chain investigations

A veterinary officer visited the implicated farm and collected 30 faecal samples, 28 of those were from the milking parlour yard, and two were from a separate area with calves. No samples were taken directly from animals. Samples were submitted to the GBRU for testing.

Food and environmental samples were collected by local Environmental Health Officers and transported in accordance with the Food Standards Agency Food Law Code of Practice [22] to the UKHSA Food, Water and Environmental Microbiology Laboratories in cold boxes at a temperature of between 0 and 8 °C and tested within 24 h of collection. All samples were examined for STEC based on the ISO/TS 13136:2012 method [23] and using a SureTect™ *E. coli* O157:H7 and STEC Screening PCR Assay (Thermofisher, Basingstoke, UK) performed according to the manufacturers instruction [24] on a QuantStudio™ 5 Real-Time PCR instrument (Applied Biosystems). Briefly, this involved enrichment in buffered peptone water, screening by real-time PCR for stx genes and then subculture onto tryptone bile glucuronic, MacConkey, and CT-SMAC agars followed by PCR of 50 suspect *E. coli* for any stx positive sample enrichments. STEC isolated from food samples were submitted to GBRU for confirmation and typing. Growing and/or processing procedures for suppliers and wholesale distributors identified in the supply chain investigation were reviewed.

### Calculation of likely period of cheese exposure and manufacture

We calculated the likely period of cheese exposure using the method described in [25], which can be summarized as the range of dates identified as follows:

- From the peak number of cases, count back the mean incubation period in days
- From the first case, count back the minimum incubation period in days
- From the last case, count back the maximum incubation period in days

We assumed a mean incubation period of 6 days and a minimum and maximum period of 3 and 7 days. We used the likely period of cheese exposure to estimate the likely date of manufacturing based on information received from the manufacturer.

## Results

### Analysis of demographic data

**Figure 1.**
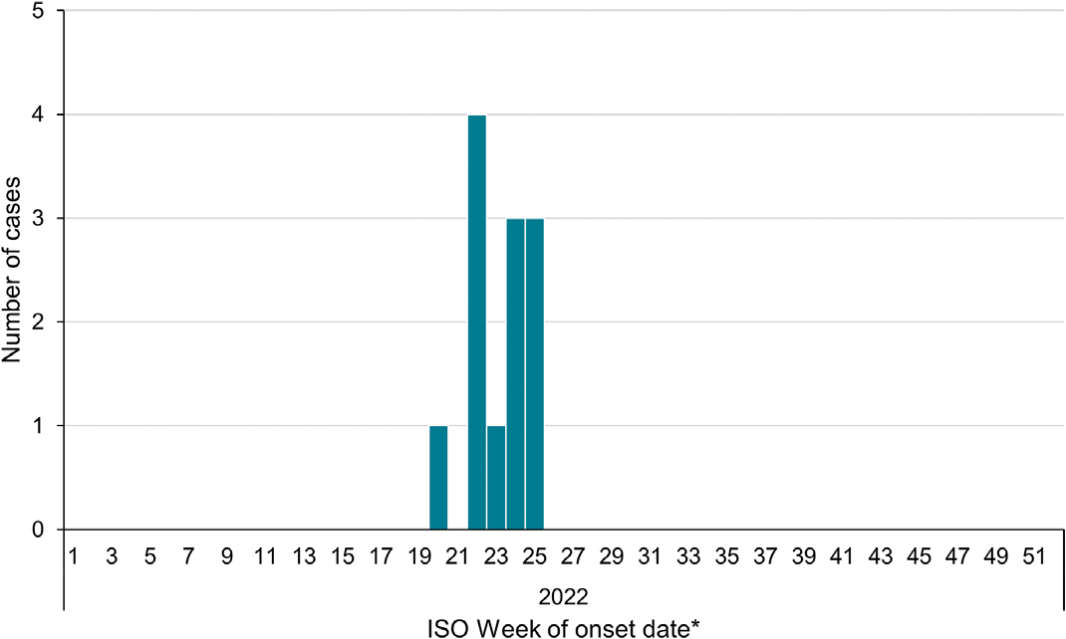
Epidemic curve of confirmed cases of STEC O103:H2 based on the onset dates, England and Wales, 2022, *N* = 12. *Sample date was used for one case where onset date was unavailable.

**Figure 2.**
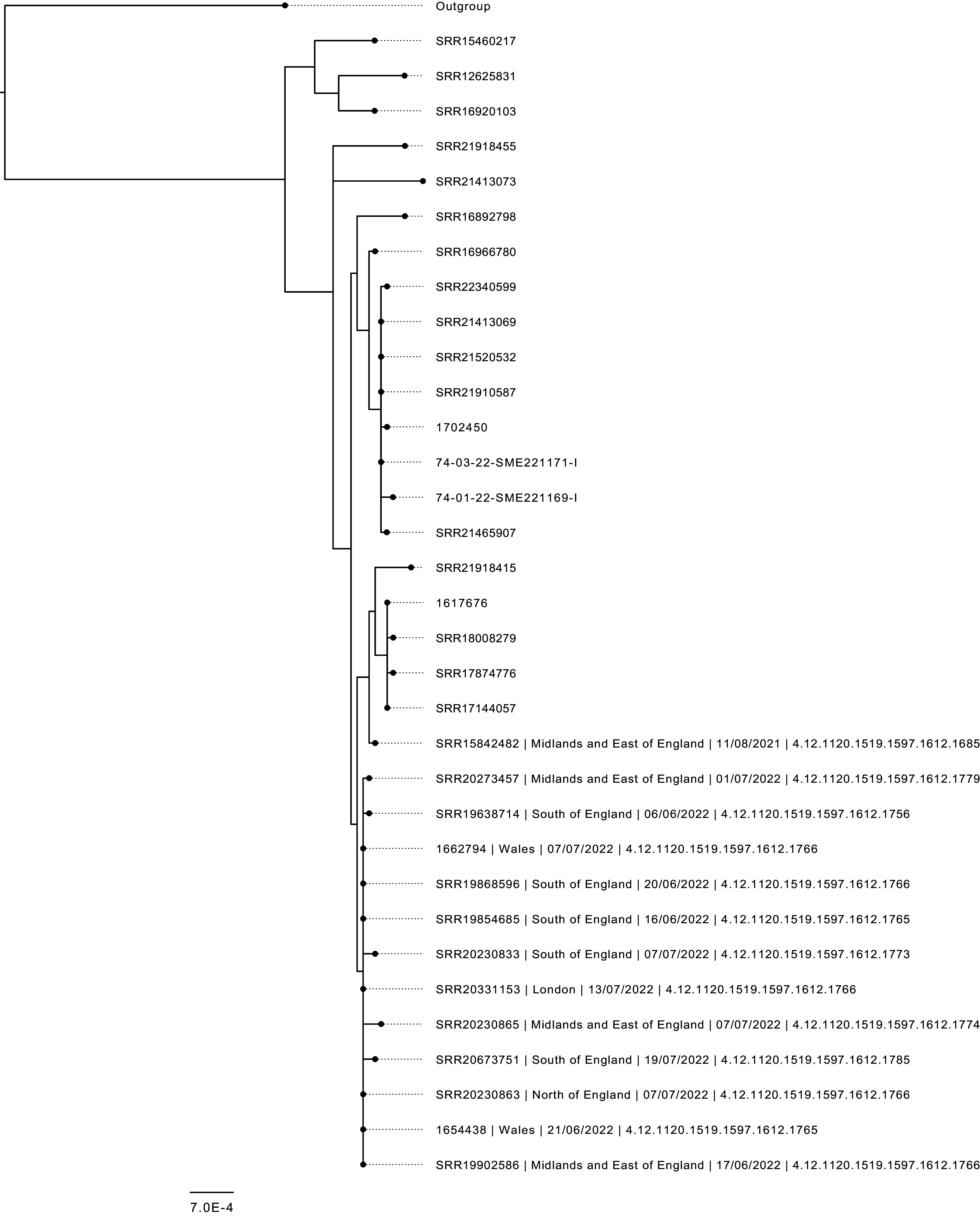
Maximum-likelihood phylogenetic tree of 5-SNP outbreak cluster in context of wider 10-SNP isolates. Outbreak cluster samples are annotated with unique identifier, region of case postcode, sample date, and SNP address. The five-SNP cluster contained one sample from 2021, which was not included in the case definitions of this outbreak cluster.

There were 12 confirmed cases reporting a date of onset (or sample date where date of onset was unavailable) between 20 May 2022 and 24 June 2022 (Figure 1). Sequences of STEC O103:H2 isolates from each case fell within two SNPs indicating they likely came from the same source (Figure 2).

**Figure 3.**
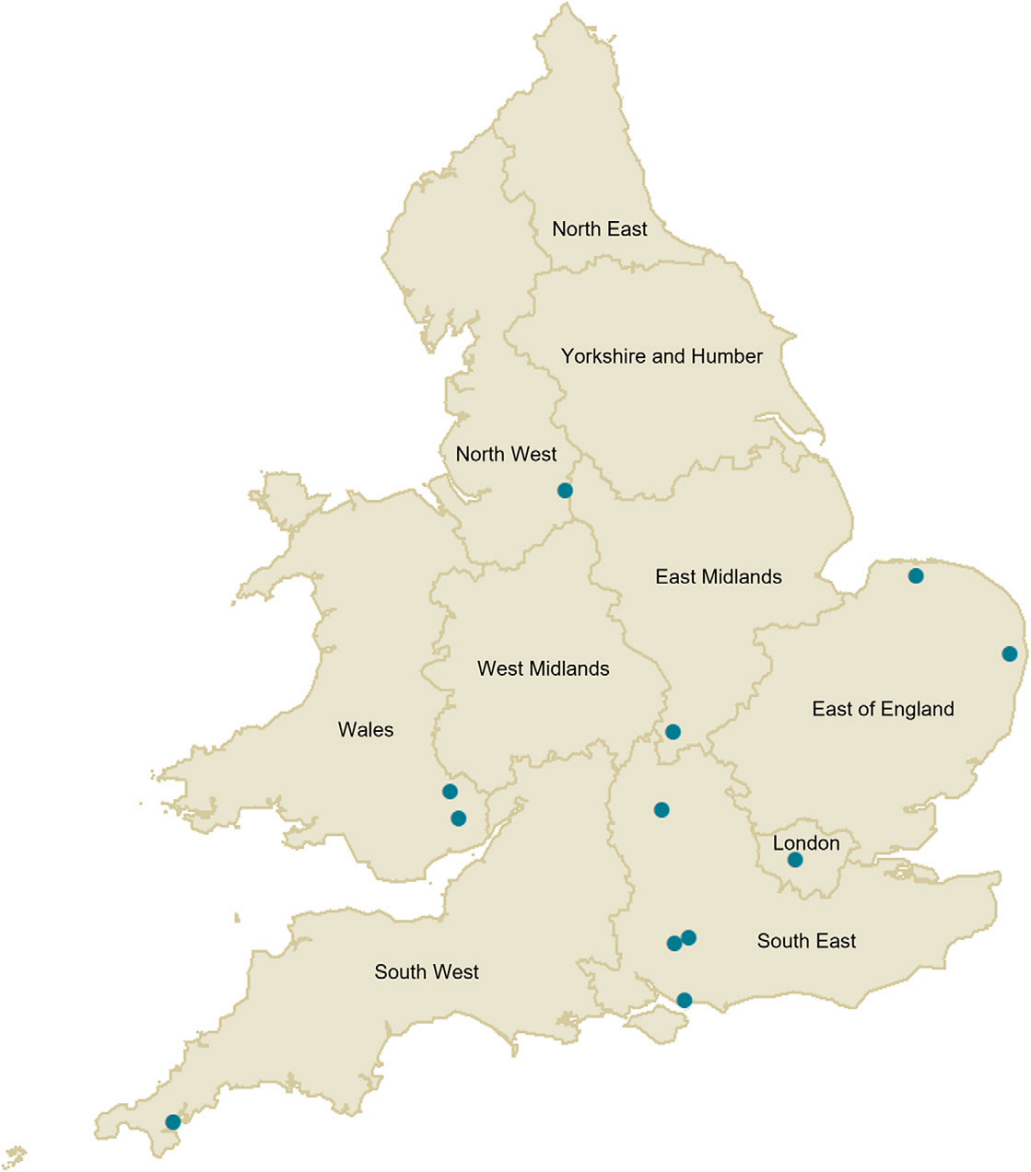
Geographic distribution of confirmed STEC O103:H2 cases during an outbreak linked by residential postcode, England and Wales, 2022, *N* = 12.

Seven cases were female, and ages of all cases ranged between 8 and 88 years (median 58 years). Ethnicity data were available for eight cases resident in England, all of which were White (White British = 7; White French = 1). Of the 12 confirmed cases, 10 were resident in England but geographically widely distributed and two were resident in Wales (Figure 3). For the English cases, the median IMD score decile was 7.5 (IQR 5–9), with 10 being the least deprived group, suggesting a relatively high socio-economic status. Data on travel were available for 11 confirmed cases, none of which reported travel outside of the United Kingdom. One case reported travelling to Scotland on a camping holiday.

### Clinical outcome data

Data on clinical presentation were available for 11 of 12 confirmed cases. Nine cases reported diarrhoea; seven reported abdominal pain; five reported bloody stools and nausea, and one reported fever and vomiting. No cases attended emergency healthcare; however, one case was hospitalized as a result of their STEC infection.

### Food histories

A total of nine confirmed cases completed an ESQ and a total of five confirmed cases completed a dairy- and salad-modified trawling questionnaire. Two cases that did not complete an ESQ did complete a trawling questionnaire. Data from the combined dataset was available for 11 of the 12 confirmed cases. Of these, nine (82%) reported soft or hard cheese consumption and eight (73%) reported eating salad leaves at home. Of those who had eaten soft cheese, six (67%) reported eating brie and of these, four (67%) reported consumption of a specific brand of unpasteurized brie-style cheese (“soft cheese A”). A further case could not recall the exact brand consumed but did report eating brie from an artisan shop that stocked the same soft cheese A; in further analysis, we assumed this case had been exposed to soft cheese A, either directly or indirectly as a result of cross contamination.

### Analytical study

Twenty-four controls were recruited, frequency matched to the 12 confirmed cases. Although food exposure data were only available for 11 of the 12 cases with various levels of completeness between them, information for all controls was kept in the analysis.

Seven food items were found to be associated with illness in the univariable analysis (any soft cheeses, any brie, soft cheese A, soft cheese B, soft cheese C, any other brand of brie not mentioned in the questionnaire, and any other salad leaves not specified in the questionnaire) (Table 1). Of these, consuming soft cheese Product A was associated with the largest odds of illness (OR 59.9, 95% CI: 2.8–1,280).

**Table 1.** Univariate analysis of the case–control study during an outbreak investigation for STEC O103:H2, England and Wales, 2022

Variable		Cases (*n* = 11)^a^	Controls (*n* = 24)	Odds ratio (OR)	95% Confidence interval (CI)	*P*-value
Composite brie variable^b^	Brie, not soft cheese A (1)	1	6	1.22	0.148, 10.1	0.006
	Only soft cheese A (2)	3	0	37.0	1.55, 886	
	Soft cheese A and other brie (3)	2	0	26.4	1.03, 678	
	No brie-style cheese (4)	3	18			
Soft cheese A^c^	Yes	5	0	59.9	2.80, 1,280	<0.001
	No	4	24			
Soft cheese B^c^	Yes	1	0	8.65	0.321, 233	0.16
	No	8	24			
Soft cheese C^c^	Yes	1	0	8.65	0.321, 233	0.16
	No	8	24			
Other brie-style cheese (not specified)	Yes	3	2	4.85	0.768, 30.6	0.09
	No	6	22			
Brie-style cheese	Yes	6	6	5.29	1.09, 25.6	0.03
	No	3	18			
Other salad leaves (not specified)	Yes	4	1	15.7	1.91, 129	0.005
	No	4	23			
Soft cheeses	Yes	6	9	3.03	0.656, 14.0	0.15
	No	3	15			

a Food history information was available for 11 out of 12 cases, but with varying completeness. Where there was no information supplied for an exposure item, the case was not included in the model; therefore, total cases will not always equal 11.

b (1) Individuals who had consumed brie that was not soft cheese A. (2) Individuals who had only consumed soft cheese A brie-style cheese. (3) Individuals who had consumed both soft cheese A brie-style cheese and other types of brie-style cheese. (4) Individuals who had not consumed any brie (this was set as the baseline).

c Only soft cheese A was produced by Producer X, soft cheese B and soft cheese C were from different brands and providers.

Because there is co-linearity between exposures to any soft cheese, any brie, and any named soft cheeses, a composite variable was created to examine them exclusively. Examining the composite soft cheese variable through univariable analysis found exposure to soft cheese A was most strongly associated with illness (OR 37.0, 95% CI: 1.55–886). Additionally, those who consumed soft cheese A and any other soft cheese were also at higher risk of illness (OR 26.4, 95% CI: 1.03–678). No association with illness was found for individuals who ate soft cheese that were not the named soft cheese of interest, and for those who did not eat any soft cheeses.

Exposures of interest from the univariable analysis were further analysed in a multivariable model for which the results are presented in Table 2. The inclusion of soft cheese B and soft cheese C were not found to be associated with illness when accounted for in the model with other salad leaves and soft cheese A. After controlling for consumption of any other salad leaves, consumption of soft cheese A remained strongly associated with illness (OR 57.5, 95% CI 3.10–1,060); as was the consumption of other salad leaves in the same model (OR 13.7, 95% CI: 1.83–103).

**Table 2.** Final multivariate model of the case–control study during an outbreak investigation for STEC O103:H2, England and Wales, 2022

Variable		Cases (*n* = 11)^a^	Controls (*n* = 24)	Adjusted odds ratio (AOR)	95% Confidence interval (CI)	*P*-value
Soft cheese A	Yes	5	0	57.5	3.10, 1,060	<0.001
	No	4	24			
Other salad leaves (not specified)	Yes	4	1	13.7	1.83, 103	0.01
	No	4	23			

a Food history information was available for 11 out of 12 cases, but with varying completeness. Where there was no information supplied for an exposure item, the case was not included in the model; therefore, total cases will not always equal 11.

### Food chain investigations

Soft cheese A was produced by a single Food Business Operator (FBO) “Producer X” which produced raw drinking milk, three different kinds of unpasteurized soft cheese including soft cheese A, and pasteurized skyr yoghurt. The FBO produced cheese on average 4 days per week and processed one batch per day (approximately 5,000 l of milk) to produce approximately 625 kg of cheese. The FBO distributed their products via wholesalers, retail premises, or directly via their onsite shop, at events, or online.

Given the earliest onset date of 20 May 2022 and the latest onset date of 24 June 2022, we calculated the likely exposure date to soft cheese A to be between 17 May and 17 June 2022 given a mean incubation period of 6 days and a minimum of 3 and maximum of 7 days. Based on additional information provided to the IMT describing how the cheese was produced, it is most likely that the contaminated batches were produced between mid-March and April 2022. The cheese producer carried out a regular sampling programme for a suite of indicator organisms as well as *E. coli* O157 and *Listeria* species. Unsatisfactory milk results and very high or high *E. coli* results in cheese were obtained during the likely production period.

A total of 30 food and environmental samples from the producer’s canteen were examined for the presence of STEC (Table 3). STEC was not detected in samples of raw milk curd or in environmental samples from the producer’s canteen. *stx* DNA was detected by PCR in 4 of 14 cheese samples tested, but isolates could not be culture confirmed, so further typing was not possible. Of note, the production date of all cheeses available for testing was later than the most likely production date of mid-March to April 2022 (Table 3).

**Table 3.** Results of STEC testing of food and environmental samples from the producer’s canteen associated with an outbreak investigation of STEC O103:H2, England, 2022

Sample date	Sampled from	Sample description	Number of samples	Number *stx* positive samples	Number of samples with confirmed STEC
27 July 22	Producer	Raw milk curd	1	0	0
27 July 22	Producer	Soft cheese A (production dates 22–30 June; use-by-dates 1–13 September)	6	3	0
10 August 22	Producer	Soft cheese A (production dates 10 June–7 July; use-by-dates 30 August–10 September)	3	1	0
03 August 22	Producer	Environmental swabs from producer’s canteen	15	0	0
10 August 22	Retail	Soft cheese A (production dates 10 June–7 July; use-by-dates 25 August–30 September	5	0	0

### Veterinary investigations

The FBO used raw milk from a closed herd of 220 cows for the production of soft cheese A. The FBO reported that it was calving season at likely time of the contamination. At the farm supplying the milk, a total of 30 cow faeces samples were taken from the milking parlour collection yard and a separate area containing calves and examined for the presence of STEC. Of these, *stx* genes were detected by PCR in three samples of which two were confirmed as O26:H11, so different from the strain detected in the human cases. Enteropathogenic *E. coli* positive for the eae gene (EPEC) were found in 11 samples (37%). Just over half (n = 16, 53%) were negative for STEC or EPEC.

## Discussion

This report describes an investigation of a small, geographically dispersed, foodborne outbreak of STEC O103:H2 detected during routine surveillance of gastrointestinal pathogens at UKHSA. Sequences of STEC O103:H2 isolates from each case fell within two SNPs indicating they came from the same source [6, 14]. Subsequent analyses of ESQs implicated unpasteurized soft cheese A from Producer X as the likely vehicle of infection. Although the outbreak strain was not detected in the cheese, a combination of genomic, epidemiological, and microbiological evidence supported this hypothesis, together providing strong evidence according to guidelines by the European Food Standards Agency [26].

We used a market research panel for the selection of our controls, a strategy that has some key advantages and limitations and has been used in multiple national outbreak investigations in the United Kingdom [20]. Market research panels allow for easy frequency matching of controls: for this outbreak, given the higher-than-average socio-economic status of cases based on their residential postcode and the artisanal nature of the cheese, we chose to match on socio-economic status as measured by IMD in addition to region and age. Although case numbers were small, we obtained a high odds ratio for soft cheese A providing a strong indication that this was the source of this outbreak. Despite our frequency matching, no controls had eaten the cheese; hence the need to use Firth’s regression for analysis. To improve confidence intervals, we could have increased controls to a higher ratio of up to 4:1, although using a 2:1 ratio still obtained a strong result. A disadvantage of using market research panels in this outbreak was that the interviewing method differed between cases and controls as a result: cases were interviewed by phone using an ESQ or modified trawling questionnaire, whereas controls self-completed an online questionnaire. In addition to the high odds ratio found for soft cheese A, we found an elevated odds ratio for salad leaves other than those specified in the questionnaire used. This finding is likely an artefact of the differences in interviewing method, with cases and controls being asked about salad products in a slightly different manner. No elevated odds were found for any named salad leaves.

Semi-selective chromogenic agar supports the growth of certain STEC serogroups, including STEC O103; however, isolation of non-O157 STEC from food and animal faeces remains challenging [6]. STEC O103 appears as a characteristic purple colony on chromogenic agar, but this culture medium is not specific for STEC. A wide variety of *E. coli* and other enterobacteriaceae found in food and animal faeces will also appear as purple colonies on chromogenic agar, and so even with semi-selective media, selecting the correct colony can be difficult [4]. The infectious dose of STEC is low (~10–100 organisms) and so contaminated food can cause infection even when the bacterial load is very low. Contaminated food often contains a heavy load of non-STEC, which further confounds detection of the target pathogen. Moreover, traditional protocols for testing food require the cold chain to maintain from sampling to testing. This does not reflect the real world, where food, including soft cheeses, is often kept at room temperature for a period of time prior to consumption.

Although there was no definitive microbiological evidence that the outbreak strain was present in the cheese, there was evidence based on the increase in bacterial load of *E. coli* that a contamination event had occurred and evidence from the PCR testing that STEC was present. The most likely production time for the contaminated cheeses was between mid-March and April: because sampling as part of the outbreak investigation did not occur until July 2022, the cheese samples tested had a much later production date: the finding that no STEC O103 was found was reassuring for the IMT that no product currently on the market was likely to cause further cases, but it did not refute our hypothesis that soft cheese A produced earlier in the year had been the source of this outbreak.

Historically, STEC surveillance in the United Kingdom focused on STEC O157 and until recently foodborne outbreaks caused by non-O157 STEC were rarely detected [27]. However, the diagnostic algorithm for detection of STEC has improved with the implementation of PCR assays for the detection of gastrointestinal pathogens at the local and regional laboratories across the United Kingdom [8]. Improvements in the epidemiological surveillance of non-O157 STEC have occurred in parallel with diagnostic improvements, and during this outbreak investigation, ESQs provided an early indication that unpasteurized soft cheese from Producer X was a likely vehicle.

Unpasteurized dairy products are a common cause of STEC outbreaks in the United Kingdom and elsewhere [28–32]. Although this was first recorded outbreak of STEC O103 in the United Kingdom, outbreaks of STEC O103 have been reported elsewhere, including outbreaks associated with unpasteurized dairy products [33–37]. FBOs in England have to comply with the Food Safety Act (1990), the Food Hygiene (England) Regulations (2013), and European Regulations to sell raw milk for drinking and to produce cheese with raw milk, which stipulate the maximum acceptable aerobic plate count of <20,000/ml and <100 coliforms/ml for raw milk and <100,000/g for cheese made with raw milk, an absence of *L. monocytogenes* in any ready-to-eat product that can support its growth, and appropriate labelling of cheese made from raw milk [38–42]. In England, the local council authorities are responsible for enforcing safe production and correct labelling of cheese made from raw milk. To verify that controlling faecal contamination of raw milk destined for raw milk cheese production is effective, it is recommended that generic *E. coli* should be below 100 cfu/ml; if between 20 and 100, an investigation should take place [43]. Routine monitoring results consistent with the type of cheese (as recommended by the Specialist Cheesemakers Assured Code of Practice) suggest that a target level of less than 100 *E. coli*/g cheese is considered achievable for most cheese types but where this is exceeded, further evidence should be provided to verify food safety [44].

Throughout the investigation, Producer X was co-operative and engaged. When the link to the unpasteurized cheese was identified, the business suspended production of unpasteurized dairy products and a review of practices, and a risk assessment was performed. Results of further microbiological testing of the unpasteurized milk were satisfactory, indicating that it was likely a one-off contamination event had occurred. The cause of the contamination remains unknown, but it is of note that this contamination event coincided with the calving season at the farm; previous studies have demonstrated a higher prevalence of STEC in calves than in adult cattle, and the risk of contamination of unpasteurized milk is likely to be higher at this time [45–47]. The calving process may also influence STEC shedding in dairy cows with increased shedding of STEC associated with first lactation, early lactation period (<30 days), and number of lactations [48, 49].

Contaminated unpasteurized milk is well-established as a cause of outbreak of STEC O157 in the United Kingdom; however, this outbreak was the first caused by non-O157 STEC and the first outbreak of STEC O103 in England [50]. The findings of this investigation provide further evidence that cattle in the United Kingdom can be colonized with STEC O103:H2 and that there is a risk of transmission to humans via contaminated food. We also highlighted the importance of using PCR to detect non-O157 STEC in ready-to-eat food, and of monitoring the presence of *E. coli* as an indicator organism. An increase in the bacterial load is a predictor of a contamination event, and a risk assessment and root cause analyses should then be carried out before the product is deemed safe for sale.

The contaminated unpasteurized cheese was a small-scale product, and the outbreak was relatively small, although given the current wide geographical spread of cases, it is likely that more cases would have been detected if more local and regional laboratories had implemented the commercial GI PCR assay. With the increasing number of laboratories using PCR, it is likely that there will be an increase in the number of non-O157 STEC outbreaks detected in the United Kingdom, and surveillance algorithms are being amended to accommodate this change in the diagnostic approach. The co-ordination and collaboration of UKHSA microbiologists, epidemiologists, and bioinformaticians with external stakeholders, including the Food Standards Agency, the Animal, and Plant Health Agency, the local authorities and the FBO were key factors in facilitating this investigation.

## Data Availability

FASTQ reads from all sequences in this study can be found at the UKHSA Pathogens BioProject at the National Center for Biotechnology Information (accession No. PRJNA315192).
